# “Fibromyalgia and quality of life: mapping the revised fibromyalgia impact questionnaire to the preference-based instruments”

**DOI:** 10.1186/s12955-017-0690-0

**Published:** 2017-05-30

**Authors:** Daniel Collado-Mateo, Gang Chen, Miguel A. Garcia-Gordillo, Angelo Iezzi, José C. Adsuar, Pedro R. Olivares, Narcis Gusi

**Affiliations:** 1 Faculty of Sport Sciences, University of Extremadura, Cáceres, Spain; 2 Centre for Health Economics, Monash University, Melbourne, Australia; 3 Department of Economics, Faculty of Economics and Business, University of Extremadura, Badajoz, Spain; 4 Department of Applied Economics, Faculty of Economics and Business, University of Murcia, Murcia, Spain; 5 Instituto de Actividad Fisica y Salud, Universidad Autonoma de Chile, Talca, Chile; 6 Instituto Superior de Educación Física, Universidad de la República, Montevideo, Uruguay

**Keywords:** EQ-5D-5 L, 15D, AQoL-8D, SF-6D

## Abstract

**Background:**

The revised version of the Fibromyalgia Impact Questionnaire (FIQR) is one of the most widely used specific questionnaires in FM studies. However, this questionnaire does not allow calculation of QALYs as it is not a preference-based measure. The aim of this study was to develop mapping algorithm which enable FIQR scores to be transformed into utility scores that can be used in the cost utility analyses.

**Methods:**

A cross-sectional survey was conducted. One hundred and 92 Spanish women with Fibromyalgia were asked to complete four general quality of life questionnaires, i.e. EQ-5D-5 L, 15D, AQoL-8D and SF-12, and one specific disease instrument, the FIQR. A direct mapping approach was adopted to derive mapping algorithms between the FIQR and each of the four multi-attribute utility (MAU) instruments. Health state utility was treated as the dependent variable in the regression analysis, whilst the FIQR score and age were predictors.

**Results:**

The mean utility scores ranged from 0.47 (AQoL-8D) to 0.69 (15D). All correlations between the FIQR total score and MAU instruments utility scores were highly significant (*p* < 0.0001) with magnitudes larger than 0.5. Although very slight differences in the mean absolute error were found between ordinary least squares (OLS) estimator and generalized linear model (GLM), models based on GLM were better for EQ-5D-5 L, AQoL-8D and 15D.

**Conclusion:**

Mapping algorithms developed in this study enable the estimation of utility values from scores in a fibromyalgia specific questionnaire.

## Background

Fibromyalgia (FM) is a chronic disease characterized by widespread pain and several associated symptoms, such as non-restorative sleep, fatigue, poor physical conditioning, impaired cognition, stiffness, depression, and balance impairment [25, 32]. These symptoms often lead a reduction in health-related quality of life (HRQoL) [9]. Although the causes of FM are still unknown, the up to date most accepted hypothesis is the sensitization of the central nervous system [14], which proposes that the cause of the high level of pain is the amplification of the sensory inputs by the central nervous system.

In Spain, approximately 4.2% of the women suffer from this disorder, whereas only 0.2% of the men are affected [19]. FM imposes significant economic burden as patients often have a high prevalence of work loss [31, 34]. Approximately 40–44% of patients are employed either full- or part-time, and the annual days missed from work are 23.2–32.5 days per year [18].

Most FM patients (83–93%) are taking at least one prescription medication, and 56–73% are taking two or more [18]. However, less than 50% of patients report being extremely or somewhat satisfied. Economic evaluations of health services are commonly used to help making decisions since resources for health care are not unlimited. These economic evaluations commonly use quality-adjusted life years (QALYs) as the unit of analysis. The estimation of the cost of obtaining an additional QALY is based on the cost-utility analysis (CUA). QALY is a single measure that comprises mortality and morbidity combining the effect on survival in years and HRQoL experienced in those years [28]. The health state utility score, which lies on a 0–1 death-full health QALY scale, is used to weight life years to reflect HRQoL. The priority is usually given to the service which results in the highest increase of QALYs for a specified cost.

The results from multi-attribute utility (MAU) questionnaires can be scored using a predetermined algorithm or weights for estimating the health utilities, which are required to calculate QALYs [6]. Clinical studies with FM patients frequently include disease specific questionnaires which have been designed to assess relevant aspects of health that are relevant for the disease, i.e. stiffness, sleep quality, fatigue, etc. The most widely used specific questionnaires in FM studies are the fibromyalgia impact questionnaire (FIQ) [3] and its revised version (FIQR) [5]. However, these two questionnaires do not allow calculation of QALYs as they are not preference-based measures. The development of mapping algorithms that enable results from disease specific questionnaires to be used in CUA is a common strategy to address the mentioned limitation [8]. This procedure has been performed in other diseases with other disease specific questionnaires, such as rheumatoid arthritis [2], idiopathic overactive bladder [12], diabetes [11], or cancer [17]. However, to our knowledge, there is no study on the development of a mapping algorithm for a FM-specific questionnaire.

The objective of the current study was to develop mapping algorithms which enable the Spanish version of FIQR scores to be transformed into utility scores that can be used in the CUA.

## Methods

### Design

A cross-sectional survey with a convenience sample was conducted between October 2014 and October 2015. Recruitment was performed at four local Spanish FM associations. The sample consisted of 192 women with FM, aged between 23 and 83 years. The inclusion criteria was: a) being a woman diagnosed with FM by a rheumatologist accordingly with the criteria of the American College of Rheumatology [33], b) being able to communicate effectively with the study staff, and (c) reading and signing the written informed consent. Data were collected through face-to-face interviews conducted by one trained and experienced researcher. Participants answered the interviews in a quiet room placed at the association’s facilities. These data were used to develop mapping algorithms between each of the four MAU instruments (EQ-5D-5 L, AQoL-8D, 15D and SF-6D) and the FIQR.

### Instruments

Four MAU instruments (Spanish version) were included in the present study. The first one is the five-level version of the world’s most widely used MAU instrument the EQ-5D. The EQ-5D-5 L [16] comprises 5 dimensions and with 5 possible levels for each dimension, defining a total of 3125 health states. The EQ-5D-5 L utility index for Spain is the result of a “crosswalk” from the EQ-5D-3 L [29]. The algorithm is available at EuroQol Group’s website (http://http://www.euroqol.org/). The EQ-5D is the most sensitive MAU instrument for measuring pain [22]. The 15D questionnaire [27] includes 15 items and 5 possible levels for each one, defining 3.05 * 10^10^ states. The AQoL-8D [23] includes 8 dimensions defining 2.37 *10^23^ states and it is the most sensitive instrument for measuring the psychosocial component [22]. The fourth MAU instrument used in the current study was the SF-6D [7], which was derived from SF-12 questionnaire and defines 18,000 health states.

The FIQR was selected as the disease specific instrument for mapping analysis. It evaluates the impact of FM addressing the limitations encountered in the FIQ while retaining the essential properties of the original instrument [5]. In this regard, the original FIQ was intended for women living in developed countries and assumed the possession of a car, a vacuum cleaner, and a washing machine. Additionally, some symptoms were included in FIQR, such as cognitive problems, tenderness, balance, and environmental sensitivity. In general terms, FIQR has replaced the original FIQ for routine use in FM related studies and clinical use. The total score of FIQR ranges from 0 to 100 and is calculated as the sum of the three domains: function domain (upper limit 30), overall impact domain (upper limit 20), and symptom domain (upper limit 50). The function domain includes nine items, the overall impact domain two items and the symptom domain includes 10 items. The validation of the Spanish version of FIQR was performed by Salgueiro et al. [24] in 2013.

### Statistical analysis

A direct mapping approach was adopted to derive mapping algorithms between the FIQR and each of the four MAU instruments. In essential, health state utility was treated as the dependent variable in the regression analysis, whilst the FIQR score and potential demographic characteristics were predictors. The regression based direct mapping is the most commonly adopted approach in the mapping literature [20]. Two models specifications were considered in this study. In the first model, the total FIQR score was the key independent variable, whilst in the second model, three domains of FIQR were key independent variables. Since participants in the current study were only women, only age was included as demographic variable. The squared terms of the dimensions and total score of FIQR were also considered in models as a way to capture non-linear effects. Independent variables were included in the model when *p*-value was lower than 0.05.

Two statistical approaches were used to calculate the two models. The first was the ordinary least squares (OLS) estimator, which is the most widely used technique [8, 20]. The second technique was the generalized linear model (GLM), which allows estimations for non-normal distributions [15]. The Gamma family with log link was chosen for the models based on the goodness of fit results.

Some pre-transformations were made before variables were entered in the model. The FIQR total/domain scores were firstly transformed onto the 0–1 scale by dividing the raw scores by the maximum total/domain scores, thus FIQR total score was divided by 100, function domain was divided by 30, overall impact domain by 20 and symptom domain was divided by 50. Regarding the EQ-5D-5 L, the original score includes negative values as it ranges from −0.654 to 1. The scale was transformed into strictly positive by adding 0.66, i.e. the whole distribution of the EQ-5D-5 L utility was moved to the right, whilst the shape of distribution remains identical. Thus the new scale ranged from 0 to 1.66. This adjustment allows the gamma family with log link to be used in the GLM estimation.

Goodness-of-fit was examined using the mean absolute error (MAE) and the root mean square error (RMSE). The lower MAE/RMSE values represents better mapping performance. To enable comparisons with other studies, the R-squared were calculated for OLS models.

An internal validation analysis was performed. The whole sample was randomly divided into five groups. In each group 80% of the sample was used to calculate the mapping algorithm and the remaining 20% was used to predict the MAU utility with the above mapping algorithm. These procedure was repeated for each of the five groups, so all groups were used as both predictor and predicted sample. MAE and RMSE were calculated in this validation analysis. This approach has been widely used in internal validation and commonly referred to as a cross-validation approach in the literature [12, 13].

All analyses were performed using Microsoft Excel 2007 software program and SPSS version 21 (IBM).

## Results

### Sample characteristics

A total of 192 women with FM aged 53.77 (10.02) years (ranged between 23 and 83 years) participated in the study. More than a half of the women (54.2%) were diagnosed between 5 and 15 years ago, and most of them (68.8%) are suffering pain and other FM symptoms since 5–25 years (see Table 1).

**Table 1:** Descriptive statistics of women with fibromyalgia (FM)

Patient characteristics	*n* = 192^a^
Mean age, years (SD)	53.77 (10.02)
Education status for participants (%)	
Non-university studies	161 (83.9)
University studies	31 (16.1)
Ocuppational status (%)	
Employee	77 (40.1)
Non employee	115 (59.9)
Mean years since clinical diagnosis of FM, *n* (%)	
< 5 years	21 (10.9)
5–15 years	104 (54.2)
15–25 years	37 (19.3)
25–55 years	14 (7.3)
Mean years since onset of symptoms, *n* (%)	
< 5 years	6 (3.1)
5–15 years	67 (34.9)
15–25 years	65 (33.9)
25–55 years	36 (18.8)
EQ-5D-5 L Utility, mean (SD)	0.49 (0.26)
AQoL-8D Utility, mean (SD)	0.47 (0.17)
15D Utility, mean (SD)	0.69 (0.12)
SF-6D Utility, mean (SD)	0.56 (0.10)
FIQR function domain, mean (SD)	15,76 (7,10)
FIQR overall impact domain, mean (SD)	9,87 (6,07)
FIQR symptom domain, mean (SD)	31,13 (10,14)
FIQR Score, mean (SD)	56.77 (20.77)

^a^ *n* = 191 for EQ-5D-5 L, *n* = 177 for AQoL-8D, *n* = 192 for 15D, *n* = 141 for SF-6D. FIQR function domain (upper limit 30); FIQR overall impact domain (upper limit 20); FIQR symptom domain (upper limit 50); FIQR Score (range 0–100)

Depending on the generic MAU instruments studied, the mean utility scores ranged from 0.47 (AQoL-8D) to 0.69 (15D). Regarding the FM-specific measure, participants had a mean (SD) FIQR score of 56.77 (20.77). According to the cut-off point between moderate and severe FM (i.e. FIQR = 59) [4, 5], on average participants had moderate to severe FM.

### Associations between instruments

Pearson correlation (R) coefficients between MAU instruments and FIQR domains/total scores are presented in Table 2. As can be seen, all correlations are highly significant (all *p* < 0.0001) with magnitudes larger than 0.5). The correlations are stronger between MAU instruments and the FIQR total score than the FIQR domain score. Among three domains, the symptom domain showed stronger association than the other two dimensions for EQ-5D-5 L, AQoL-8D, and 15D, whereas for SF-6D, the overall impact domain showed the highest R value. Overall speaking, among four MAU instruments, the correlation between EQ-5D-5 L and FIQR was the strongest.

**Table 2:** Pearson correlations between the generic multi-attribute utility instruments and the FIQR scores

	EQ-5D-5 L utility *n* = 191	AQoL-8D utility *n* = 177	15D utility *n* = 192	SF-6D utility *n* = 141
FIQR function domain	–.668**	−.523**	−.592**	−.599**
FIQR overall impact domain	−.653**	−.598**	−.593**	−.605**
FIQR symptom domain	−.686**	−.668**	−.673**	−.595**
FIQR Score	−.749**	−.682**	−.704**	−.684**

**All correlations were statistically significant, *P* < 0.0001

### Mapping results

Table 3 presents the goodness-of-fit for the four analyzed MAU instruments using full data. Two panels can be identified: Panel A shows the model with FIQR, squared FIQR and age, while Panel B includes the model with the three dimensions of FIQR, the three squared-dimensions of FIQR and age.

**Table 3:** Goodness-of-fit results for mapping FIQR scores onto multi attribute utilities

PANEL A								
Instruments	*N*	Method	Mean Utility	Min Utility	Max Utility	MAE	RMSE	R-squared
AQoL-8D	177	Observed	0.465	0.155	0.945	-	-	
		OLS	0.465	0.213	0.778	0.097	0.124	0.465
		Gamma GLM	0.465	0.267	0.866	0.095	0.123	
15D	192	Observed	0.688	0.391	1.000	-	-	
		OLS	0.688	0.487	0.931	0.069	0.087	0.507
		Gamma GLM	0.688	0.510	0.967	0.069	0.087	
EQ-5D-5 L	191	Observed	0.490	−0.480	1.000	-	-	
		OLS	0.490	−0.079	0.819	0.138	0.174	0.579
		Gamma GLM	0.490	0.011	0.870	0.137	0.174	
SF-6D	141	Observed	0.561	0.345	1.000	-	-	
		OLS	0.561	0.411	0.737	0.063	0.079	0.468
		Gamma GLM	0.561	0.428	0.756	0.063	0.079	
PANEL B								
Instruments	*N*	Method	Mean Utility	Min Utility	Max Utility	MAE	RMSE	R-squared
AQoL-8D	177	Observed	0.465	0.155	0.945	-	-	
		OLS	0.465	0.228	0.787	0.097	0.123	0.480
		Gamma GLM	0.465	0.275	0.876	0.096	0.122	
15D	192	Observed	0.688	0.391	1.000	-	-	
		OLS	0.688	0.507	0.950	0.071	0.088	0.492
		Gamma GLM	0.688	0.511	0.979	0.068	0.086	
EQ-5D-5 L	191	Observed	0.490	−0.480	1.000	-	-	
		OLS	0.490	−0.081	0.836	0.138	0.175	0.573
		Gamma GLM	0.490	0.010	0.890	0.137	0.176	
SF-6D	141	Observed	0.561	0.345	1.000	-	-	
		OLS	0.561	0.410	0.737	0.063	0.079	0.472
		Gamma GLM	0.561	0.427	0.756	0.063	0.079	

Panel A: FIQR, Squared-FIQR, Age

Panel B: FIQR function domain; FIQR overall impact domain; FIQR symptom domain; Squared FIQR function domain; Squared FIQR overall impact domain; Squared FIQR symptom domain; Age. Gamma GLM: Generalized linear model Gamma log-link

The predicted mean utilities using OLS or GLM were always identical to the observed means. Regarding the predicted utility range all models tended to under predict the highest utility and over predict the lowest utility. Highest discrepancies between the predicted minimum and the observed minimum were found for EQ-5D, while the lowest differences were observed in SF-6D. OLS always predicted a minimum closer to the observed one compared to GLM. Both OLS and GLM tended to under predict the maximum scores, with highest discrepancies in SF-6D and good accuracy for 15D. The performance of GLM predicting maximum scores were much better than OLS, especially in the AQoL-8D model.

MAE and RMSE were calculated as key goodness-of-fit measures. The MAE was lowest for SF-6D (0.063) and highest for EQ-5D-5 L (0.137). The MAE for AQoL-8D and 15D was 0.097 and 0.069 respectively. Although very slight differences in the MAE were found between OLS and GLM, models based on GLM were better for EQ-5D-5 L, AQoL-8D and 15D. MAE values were identical for SF-6D. Similar results were observed for RMSE.

The R-squared statistics ranged from 0.465 for AQoL-8D in the Panel A to 0.579 for EQ-5D-5 L in the Panel A. Judging on the R-squared, the mapping performance of the AQoL-8D model was better in Panel B, i.e. using FIQR domain scores instead of the total score as key predictors. This conclusion is also applicable to the SF-6D. On the other hand, EQ-5D-5 L and 15D showed higher R-squared statistic values in the Panel A using total score of the FIQR.

Table 4 shows the goodness-of-fit from the validation analysis. The MAE and RMSE were similar to those reported in Table 3. Highest discrepancies were observed in the Panel B of SF-6D, where MAE was enhanced from 0.063 in Table 3 to 0.066 and 0.067 in Table 4 for OLS and GLM respectively. RMSE was also increased in the validation analysis from 0.079 to 0.084. The best model for predicting utilities from the FIQR differs depending on the MAU instruments. The best model for AQoL-8D is the GLM as the MAE and RMSE were lower compared to OLS. Performance with Panel A or Panel B was very close: MAE was slightly lower in Panel A and RMSE was slightly lower in Panel B. Range was also very close. The validation analysis showed slightly lower MAE for Panel A (0.096 vs 0.097) and the same RMSE, but the predicted range was much better in Panel B, as the maximum was 0.901 while it was 0.837 in Panel A. Therefore, both models, using FIQR score and dimensions can be accepted. The best model for 15D is the GLM using FIQR dimensions, as the MAE and RMSE were slightly lower. As happened with AQoL-8D, all models seemed to be valid and differences between them were very small. The performance of the model for EQ-5D-5 L and SF-6D were also similar for both types of statistical approach and panels. No clear differences were observed in terms of MAE and RMSE, and the range was slightly better for GLM in the Panel B. Validation analysis confirmed this tendency.

**Table 4:** Goodness-of-fit results from validation analysis

PANEL A							
Instruments	*N*	Method	Mean Utility	Min Utility	Max Utility	MAE	RMSE
AQoL-8D	177	Observed	0.465	0.155	0.945	-	-
		OLS	0.465	0.214	0.751	0.099	0.126
		Gamma GLM	0.465	0.269	0.837	0.096	0.124
15D	192	Observed	0.688	0.391	1.000	-	-
		OLS	0.688	0.484	0.927	0.070	0.087
		Gamma GLM	0.688	0.511	0.965	0.069	0.087
EQ-5D-5 L	191	Observed	0.490	−0.480	1.000	-	-
		OLS	0.490	−0.076	0.814	0.138	0.174
		Gamma GLM	0.489	0.016	0.871	0.137	0.175
SF-6D	141	Observed	0.561	0.345	1.000	-	-
		OLS	0.560	0.415	0.739	0.064	0.081
		Gamma GLM	0.560	0.431	0.756	0.064	0.081
PANEL B							
Instruments	*N*	Method	Mean Utility	Min Utility	Max Utility	MAE	RMSE
AQoL-8D	177	Observed	0.465	0.155	0.945	-	-
		OLS	0.465	0.218	0.808	0.098	0.124
		Gamma GLM	0.465	0.271	0.901	0.097	0.124
15D	192	Observed	0.688	0.391	1.000	-	-
		OLS	0.689	0.498	0.942	0.072	0.089
		Gamma GLM	0.689	0.521	0.985	0.071	0.089
EQ-5D-5 L	191	Observed	0.490	−0.480	1.000	-	-
		OLS	0.491	−0.085	0.843	0.138	0.174
		Gamma GLM	0.490	0.014	0.891	0.137	0.175
SF-6D	141	Observed	0.561	0.345	1.000	-	-
		OLS	0.559	0.411	0.743	0.066	0.084
		Gamma GLM	0.559	0.428	0.761	0.067	0.084

Validation analysis dividing the sample into 5 groups (80% as predictor and 20% as predicted)

Panel A: FIQR, Squared-FIQR, Age

Panel B: FIQR function domain; FIQR overall impact domain; FIQR symptom domain; Squared FIQR function domain; Squared FIQR overall impact domain; Squared FIQR symptom domain; Age

Gamma GLM: Generalized linear model Gamma log-link

Table 5 reports the detailed significant regression coefficients for each mapping algorithm. Age was only significant in predicting 15D utility. Results from Table 5 can be utilized to predict health state utility in case only FIQR score is available. For example, the optimal mapping algorithm from FIQR domain scores to the SF-6D utility is derived based on the OLS estimator, and can be written as:

**Table 5:** Mapping equations from FIQR score to multi-attribute utility instruments

	AQoL-8D		15D		EQ-5D-5 L		SF-6D	
	Coeff.	SE	Coeff.	SE	Coeff.	SE	Coeff.	SE
PANEL A								
OLS								
Constant	.778	.027***	.997	.039***	1.479	.024***	.794	.022***
FIQR/100	−.564	.046***	−.420	.030***			−.383	.035***
Age			−.001	.001*				
Squared-FIQR					−.898	.056***		
GLM								
Constant	**−.144**	.060*	.068	.058	**.425**	.025***	−.179	.039***
FIQR/100	**-1.175**	.101***	−.600	.046***			−.670	.061***
Age			−.002	.001*				
Squared-FIQR					**−.824**	.058***		
PANEL B								
OLS								
Constant	.787	.031***	**.950**	.021***	1.496	.027***	**.786**	.025***
Age								
FIQR_D1			**−.138**	.036***			**−.137**	.043**
FIQR_D2	−.144	.043**					**−.089**	.034**
FIQR_D3	−.415	.065***	**−.305**	.042***			**−.149**	.049**
Squared FIQR_D1					−.335	.081***		
Squared FIQR_D2					−.167	.064**		
Squared FIQR_D3					−.415	.084***		
GLM								
Constant	−.132	.068*	.078	.059	0.438	.028***	−.192	.044***
Age			−.002	.001*				
FIQR_D1			−.156	.058**			−.241	.075**
FIQR_D2	−.318	.096**	−.096	.048*			−.153	.059**
FIQR_D3	−.840	.147***	−.359	.070***			−.264	.085**
Squared FIQR_D1					−.304	.081***		
Squared FIQ_D2					−.167	.065**		
Squared FIQR_D3					−.368	.086***		

Coeff. and SE: Beta and Standard error of significant variables (*p < 0.05, **p < 0.01; ***p < 0.001) in the equations

Panel A: FIQR, Squared-FIQR, Age

Panel B: FIQR function domain (FIQR_D1); FIQR overall impact domain (FIQR_D2); FIQR symptom domain (FIQR_D2); Squared FIQR function domain; Squared FIQR overall impact domain; Squared FIQR symptom domain; Age

Gamma GLM: Generalized linear model Gamma log-link. EQ-5D was pre-transformed as EQ-5D + 0.66 in order to calculate the Gamma GLM with all scores (including negative values). It was post-transformed as EQ-5D – 0.66

Coefficients with best fitting for equations are in bold

SF-6D utility = 0.786–0.137*FIQR_D1–0.089*FIQR_D2–0.149*FIQR_D3where FIQR_Di (*i* = 1, 2, 3) are pre-adjusted domain scores; FIQR_D1 is the function domain, FIQR_D2 is the overall impact domain, and FIQR_D3 is the symptom domain.

## Discussion

The funding of drugs and medical services in health schemes is increasingly contingent upon the successful outcome of an economic evaluation. When HRQoL is an important outcome regulatory authorities (e.g. the National Institute for Health and Care Excellence in the United Kingdom) generally recommend or require the use of CUA, which implies the need to use health state utility as the outcome measure. This study investigated four generic MAU and one disease-specific instruments on patients with FM. In addition, based on this unique dataset, a set of mapping algorithms were reported so that health state utilities can be predicted when only FIQR is administrated. This study is the first enabling the estimation of utility values from scores in a FM specific questionnaire.

The severity of symptoms of women with FM who participated in the current study was very similar to the previous study where FIQR was validated [5]. In that study, mean score ± SD was 56.6 ± 20.0, while results in Table 1 of this manuscript showed a mean score ± SD of 56.77 ± 20.77. Therefore, the patient sample used to derive/validate mapping algorithm covers the most commonly observed FM severity in clinical practice.

There were relevant differences between utility scores of the four analyzed MAU instruments. Among them, the EQ-5D-5 L was found to have the strongest correlation (−0.749) with FM-specific measure. This is probably because the EQ-5D-5 L primarily reflect pain and physical function (Richardson et al. [22]). Since pain is the main symptom of FM and the impact of the disease on physical health may be even higher than the impact on psychological health [26], it seems reasonable that this questionnaire presented higher correlation coefficient (−0.749) when it was correlated with FIQR. On the other hand, the mean utility score of AQoL-8D was the lowest among four MAU instruments. This contrasts with the results for healthy population, when lowest scores are often observed for SF-6D. That could indicate that social component has large impact in the HRQoL of women with FM as it was reported in previous studies [1, 35].

R-squared coefficient is often about 0.5 when mapping is performed from a generic onto generic preference-based questionnaires. However, this value is commonly reduced to 0.2–0.4 when mapping is performed from disease specific to generic questionnaires [8]. The R-squared in the current study ranged from 0.465 for AQoL in the Panel A to 0.579 for EQ-5D-5 L in the Panel B. This high scores indicate strong relation between FIQR and HRQoL, and highlight the relevance of pain and physical function in the utility score of EQ-5D-5 L.

The study has several limitations. Firstly, the range of the predicted utilities was narrower than the observed utilities. This is a commonly reported limitation in mapping literature [11, 12, 30]. This range was particularly narrow in EQ-5D-5 L and SF-6D. As can be seen in the supplemental file, the distribution of these questionnaires in the sample of the current study may have increased this problem. Specifically, one women scored −0.480 in EQ-5D-5 L, while the second lowest score in this questionnaire was −0.133. Therefore, if this anomalous value was removed, the observed minimum would be −0.133, which is similar to the predicted minimum (−0.08 using OLS and 0.01 using GLM). Similarly, the SF-6D utility score of one woman was 1, while the second best utility score in this instrument was 0.86. Again, the narrow range problem is largely caused by an extreme value, as the predicted maximum was 0.756 (GLM model). Therefore, it should be noticed that the model performance is reduced for extreme values. Although there are previous mapping studies with smaller sample size [12, 20], this may represent the second limitation. Another limitation regarding the sample is the absence of males. This is important because the mapping algorithms from the current study should only be applied to women and not men. Given the scarce of men diagnosed with FM in Spain and the large sex differences among FM patients [10, 21], studies are commonly focused in one of the two genders. However, further studies are encouraged to develop a mapping algorithm for men suffering from FM. The fourth limitation is the validation of the model using internal data. Although the absence of an external validation is not uncommon, external validation is strongly recommended. Finally, mapping onto the crosswalk version EQ-5D-5 L tariff means there is a “double-mapping”, however there is no other Spanish EQ-5D-5 L tariff available for use.

Despite these limitations, the R-squared and the goodness-of-fit measures presented values as good as or better than the reported in previous mapping studies [8, 20]. The results indicate that FIQR con be mapped onto EQ-5D-5 L, 15D, AQoL-8D and SF-6D with good precision in a FM sample .

## Conclusion

Mapping algorithms developed in this study enable the estimation of utility values from scores in a FM specific questionnaire. FIQR con be mapped onto EQ-5D-5 L, 15D, AQoL-8D and SF-6D with good precision in this population. Therefore, the current study enables CUA using data from the most used FM specific disease questionnaire.
